# Frailty Improvement by Multicomponent Drug, Ninjin’Yoeito, in Mild Cognitive Impairment and Mild Alzheimer’s Disease Patients: An Open-Label Exploratory Study (FRAMINGO)

**DOI:** 10.3233/ADR-220074

**Published:** 2023-02-01

**Authors:** Kazunori Okahara, Makoto Ohsawa, Ayaka Haruta-Tsukamoto, Ryoei Miyoshi, Hideki Funahashi, Yasuhiro Fukutani, Setsuko Makita, Hisae Matsuo, Yasushi Ishida

**Affiliations:** a Keimei Memorial Hospital, Higashimorokata, Miyazaki, Japan; b Oido Clinic, Isesaki-city, Gunma, Japan; c Department of Psychiatry, Faculty of Medicine, University of Miyazaki, Miyazaki-city, Miyazaki, Japan; d Heartopia Miyoshi Clinic, Miyazaki-city, Miyazaki, Japan; e Center for Health Sciences and Counseling, Kyushu University, Nishi-ku, Fukuoka, Japan

**Keywords:** Alzheimer’s disease, anorexia, dementia, fatigue, frailty, Kampo, mild cognitive impairment, Ninjin’yoeito

## Abstract

**Background:**

Alzheimer’s disease (AD) and dementia have increasingly been conceived of as “complex diseases of aging”, determined by multiple, simultaneous, interacting pathophysiological processes. The condition known as frailty is a phenotype of aging and its comprehensive pathophysiology is thought to be closely related to the incidence of mild cognitive impairment (MCI) and the exacerbation of dementia.

**Objective:**

This study aimed to investigate the effect of the multicomponent drug, ninjin’yoeito (NYT), on frailty in MCI and mild AD patients.

**Methods:**

This study was an open-label trial. A total of 14 patients, including 9 with MCI and 5 with mild AD, were enrolled. Among them, 11 were frail while 3 were prefrail. NYT (6–9 g/day) was administered orally for 24 weeks, and assessments were carried out at baseline (week 0), and at 4, 8, 16, and 24 weeks.

**Results:**

In the primary endpoint, significant early improvements were observed in the anorexia scores according to the Neuropsychiatric Inventory after four weeks of treatment with NYT. The Cardiovascular Health Study score was significantly improved, and no frailty was observed after 24 weeks. The fatigue visual analog scale scores also significantly improved. The Clinical Dementia Rating and the Montreal Cognitive Assessment scores remained at baseline levels during the NYT treatment period.

**Conclusion:**

The results suggest that NYT may be effective in the treatment of frailty, especially for anorexia and fatigue, in both MCI and mild AD patients, which would be beneficial for the prognosis of dementia.

## INTRODUCTION

Mild cognitive impairment (MCI) is a condition that puts individuals at high risk of developing dementia and is recognized as a precursor to dementia, particularly Alzheimer’s disease (AD). Individuals with MCI have a more rapid rate of cognitive decline than those without cognitive impairment [1]. Dementia, including AD, presents with behavioral and psychological symptoms of dementia (BPSD), as well as core symptoms such as cognitive impairment. BPSD may manifest with agitation, aberrant motor behavior, anxiety, elation, irritability, depression, apathy, disinhibition, delusions, hallucinations, and sleep or appetite impairments. These symptoms have adverse consequences for patients and caregivers, such as greater impairment in activities of daily living, worsening quality of life, and earlier institutionalization [2].

Anorexia is a problematic symptom of BPSD because it results in deterioration of the nutritional status of patients which negatively affects their general condition. Approximately 20% of patients with AD have anorexia [3], and weight loss is often observed in the late life period several years before the onset of dementia [4] or MCI [5]; therefore, anorexia may contribute to the onset and exacerbation of dementia. Recently, a novel nutrition-related multivariate biomarker for MCI, which may be associated with malnutrition, has also been identified [6].

Frailty is a measure of physiological vulnerability and is characterized by the accumulation of health deficits over time [7]. The five phenotypic components of physical frailty are the following: 1) unintentional weight loss, 2) low grip strength, 3) self-reported exhaustion, 4) slow walking speed, and 5) physical inactivity [8]. Malnutrition and weight loss due to anorexia lead to sarcopenia, which then induces exhaustion, a decline in physical function (low grip strength and slow walking speed), low physical activity, and low energy expenditure. This further promotes anorexia and malnutrition, forming a negative spiral, described as the cycle of frailty [9]. Longitudinal changes in frailty have been associated with adverse health outcomes including disease-specific morbidity [10], mortality [11], institutionalization, and disability [12]. Frailty is also an established risk factor for cognitive decline [13] and dementia [14] and is associated with incident MCI [15]. Thus, AD and dementia have increasingly been conceived of as “complex diseases of aging” determined by multiple, simultaneous, interacting pathophysiological processes [16].

Ninjin’yoeito (NYT) is a traditional Japanese medicine (Kampo medicine) and a multi-component formulation composed of twelve crude drugs. It has been approved by the authorities in Japan as a prescription drug and is indicated for constitutional decline from disease, fatigue, anorexia, perspiration during sleep, cold limbs, and anemia. In 2007, Seiwa et al. reported that NYT reversed age-induced demyelination, leading to an acceleration of axonal remyelination in the brain, and indicated its role in cognitive function [17]. Kudoh et al. showed that NYT improved cognitive function and depression in patients with AD when added to donepezil treatment [18]. Furthermore, the potential efficacy of NYT against frailty by improving grip strength [19], reducing fatigue, and anorexia [20] in older people has been reported.

Our previous open-label study involving patients with AD indicated that NYT improved anorexia and apathy [21]. However, no trials have examined the effects of NYT on frailty in MCI. Thus, we performed an open-label exploratory study of FRAil patients with Mild cognitive Impairment or mild dementia to investigate the efficacy and safety of Ninjin’yoeito in Geriatric Outpatient clinics (FRAMINGO).

## MATERIALS AND METHODS

### Study design

This was an open-label trial to test the efficacy and safety of NYT on anorexia in prefrail or frail patients with MCI or mild dementia over a 24-week treatment period. The subjects visited the study site at screening (four weeks before starting treatment), baseline (week 0), and at 4, 8, 16, and 24 weeks. The data were collected from July 2019 to December 2021.

### Ethics policies and patient permissions

This study was conducted according to the principles of the Declaration of Helsinki and followed the Clinical Trials Act enforced by Japanese authorities. The study protocol was approved by the certified review board of the University of Miyazaki (CRB7180007). Three sites participated in this trial after obtaining approval from the CRB. The study was registered in the Japan Registry of Clinical Trials (registration ID number: jRCTs071190011). Informed consent to participate in the study was obtained from each subject or legally authorized representative, and the study partner cooperated with the efficacy evaluation.

### Subjects

The following individuals were eligible if they met all the inclusion criteria: individuals with MCI, as defined by the criteria set out by the Diagnostic and Statistical Manual of Mental Disorders (Fifth Edition) (DSM-5) [22], or probable/possible AD, AD with cerebrovascular disease, and mixed dementia as defined by the National Institute on Aging-Alzheimer’s Association criteria [23]. The main inclusion criteria were age ≥55 years, outpatients, Neuropsychiatric Inventory (NPI) subcategory scores for ‘anorexia’ ≥3 points, prefrailty or frailty defined as the Japanese version of the Cardiovascular Health Study (J-CHS) criteria, and a global Clinical Dementia Rating (CDR) score of 0.5 or 1.0.

Individuals were excluded if they had severe agitation/aggression, malignant tumor, or other life-threatening diseases, and had major depression, bipolar disorder, schizophrenia, alcoholism, or drug addiction (described in DSM-5) within the past two years. Individuals were also excluded if they had taken medicines containing Polygala Root, Citrus Unshiu Peel, or Kampo medicine, which had indications for anorexia, fatigue, weakness after illness, and upper gastrointestinal disorders such as gastritis, within the past 4 weeks. There were two categories of concomitant drugs administered during the study period. In category 1, continued stable use during the treatment period was allowed for therapeutic agents for dementia (cholinesterase inhibitors) started within the previous 24 weeks, and other effective agents for cognitive decline, frailty, and peptic ulcer started within the previous 4 weeks. For category 2, the use of hypnotics, psychotropic agents, anticonvulsants, effective agents for anorexia, and first-generation antihistamines as rescue drugs were allowed when complications worsened during the study period; however, the dose and frequency of use were kept to a minimum.

### Study medication

TSUMURA Ninjin’yoeito Extract Granules for Ethical Use (TJ-108; Tsumura & Co., Tokyo, Japan) is composed of twelve crude drugs (Rehmannia Root, Japanese Angelica Root, Atractylodes Rhizome, Poria Sclerotium, Ginseng, Cinnamon Bark, Polygala Root, Peony Root, Citrus Unshiu Peel, Astragalus Root, Glycyrrhiza, and Schisandra Fruit), and was administered three times daily (3 g each time, 9 g/day) during the 24-week treatment period. The dose of NYT could be reduced to 6 g/day administered twice daily, depending on the subject’s age, body weight, and response to the treatment (e.g., adverse events). For the assessment of adverse events, we used the package insert of NYT and medical opinion to judge whether there was a causal relationship between the treatment and the adverse reaction. Approval was received from the CRB.

### Outcome measures

The NPI score was derived as the product of the frequency ratings for the four items and severity ratings for the three items. The NPI subcategory comprises a maximum 12-point scale, with higher scores indicating greater impairment [24]. The primary outcome measure for this trial was the 24-week change in score for ‘anorexia’, according to the NPI subcategory for eating disturbances. Secondary outcome measures included changes in J-CHS score [25]; body composition (body weight, lean body mass, and fat mass) evaluated by InBody470 (InBody Japan, Tokyo, Japan); fatigue visual analog scale (fatigue VAS); blood nutrition index, controlling nutritional status (CONUT) score using serum albumin level, total lymphocyte count, and total cholesterol level [26]; NPI subcategory score for ‘depression’ and ‘apathy’; global CDR and CDR-Sum of Boxes (CDR-SB) [27, 28]; and the Japanese version of the Montreal Cognitive Assessment (MoCA-J) [29, 30] total score and safety assessments by week 24. In addition, demographic data including brain magnetic resonance imaging (MRI) or computed tomography (CT), medical history, and laboratory tests were examined.

### Statistical analysis

The analysis set for the efficacy evaluation included all patients who met eligibility requirements and received treatment. The changes in the period from week 0 to weeks 4, 8, 16, and 24 of the study treatment were evaluated by calculation of summary statistics and Wilcoxon signed rank test or two-tailed paired *t*-test. *p* values <0.05 were considered statistically significant. For nonparametric data, the Wilcoxon signed rank test was used and presented as medians and interquartile ranges. For normally distributed data, a paired t-test was used and presented as mean±standard deviation (SD). No adjustments were carried out for multiple testing. The R Software (version 3.4.0) was used for statistical analyses.

## RESULTS

### Subject flow, protocol alteration, and demographics

Data from 14 patients, including 9 with MCI and 5 with mild AD, were analyzed to assess the efficacy and safety of NYT treatment. None of the patients withdrew from the trial due to adverse events. One patient could not be assessed at the final visit due to a fracture; therefore, data at 24 weeks were missing. The dose of NYT in one patient was reduced to 6 g/day at week 4 due to the occurrence of an adverse event (edema). The other patients continued to receive 9 g/day NYT up to week 24 (data not shown). During the course of the trial, the protocol was altered to accelerate subject enrollment by changing concomitant drugs for peptic ulcers from Category 2 to Category 1. The demographic and clinical characteristics of the patients at baseline are shown in Table 1. The average age of the patients was 80.0±6.2 years. A total of 11 patients with frailty and 3 with prefrailty according to the J-CHS criteria were enrolled. Among them, 7 patients had white-matter lesions. The global CDR and MoCA-J total scores were 0.5 (0.5-0.5) and 18.0 (14.3–20.8) at baseline, respectively.

**Table 1:** Baseline characteristics and demographics of the subjects

Variable	*n* = 14
Age, y	80.0±6.2
Gender, M/F	4/10
MCI/mild AD	9/5
Body weight, kg	47.5±9.5
BMI, kg/m^2^	21.0±3.1
Frailty/prefrailty	11/3
Global CDR	0.5 (0.5-0.5)
MoCA-J total score	18.0 (14.3–20.8)
White matter lesion, absence/presence	7/7
Complications	8
Concomitant treatment	12
Allergy to medicines	0
Serum potassium, mEq/L	4.3±0.5

Data are presented as the mean±SD or median with quartiles in parentheses. Frailty/prefrailty was assessed using the J-CHS criteria, and white matter lesions were judged by brain MRI or CT. MCI, mild cognitive impairment; AD, Alzheimer’s disease; CDR, Clinical Dementia Rating; MoCA, Montreal Cognitive Assessment; Complications, number of patients with complications; Concomitant treatment, patients administered medications for complications.

### Primary outcome measures

**Fig. 1:**
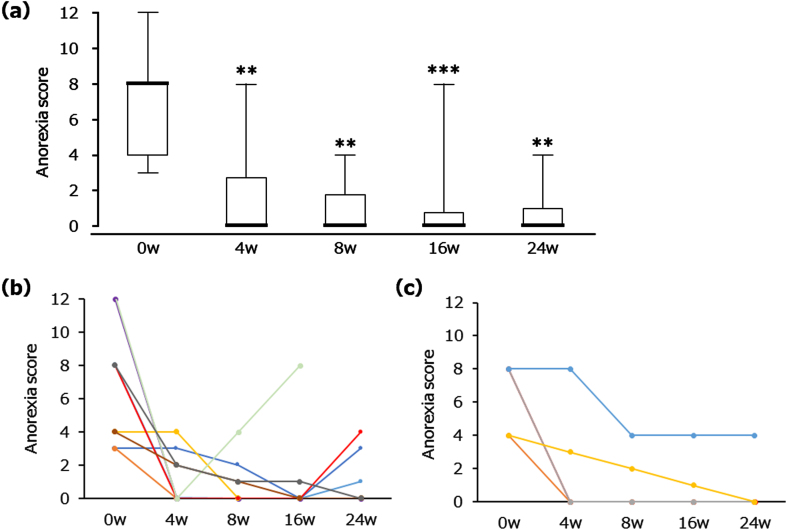
Changes in the score for ‘anorexia’ in the NPI subcategory for eating disturbances after NYT treatment. Box plot (a) showing median, quartiles, and bars indicating the maximum and minimum values; *n* = 14 (0, 4, 8 and 16 weeks (w)), *n* = 13 (24 w). ^**^p < 0.01, ^***^p < 0.001 versus 0 w (Wilcoxon signed-rank test). Line graphs showing all individual data in MCI (b) and mild AD (c), separately. *n* = 9 (MCI), *n* = 5 (mild AD). The score was derived from the product of the frequency ratings for four items and severity ratings for three items (maximum 12-point scale).

The primary outcome measures are shown in Fig. 1. Treatment with NYT resulted in a significant reduction in the score for ‘anorexia’, according to the NPI subcategory for eating disturbances, by week 4 (Fig. 1a). The median and quartiles were as follows. Baseline: 8.0 [4.0–8.0]; 4 weeks: 0.0 [0.0–2.8], *p* < 0.01; 8 weeks: 0.0 [0.0–1.8], *p* < 0.01; 16 weeks: 0.0 [0.0–0.8], *p* < 0.001; 24 weeks: 0.0 [0.0–1.0], *p* < 0.01. The mean and SD of reference data were the following. Baseline: 6.7±3.1; 4 weeks: 1.6±2.3; 8 weeks: 1.0±1.5; 16 weeks: 1.0±2.3; 24 weeks: 0.9±1.6. Figure 1b and 1c demonstrate the changes in individual anorexia score for MCI and mild AD patients, respectively. Anorexia scores were reduced in all patients without diagnostic distinction as to MCI or mild AD status.

### Secondary outcome measures

**Fig. 2:**
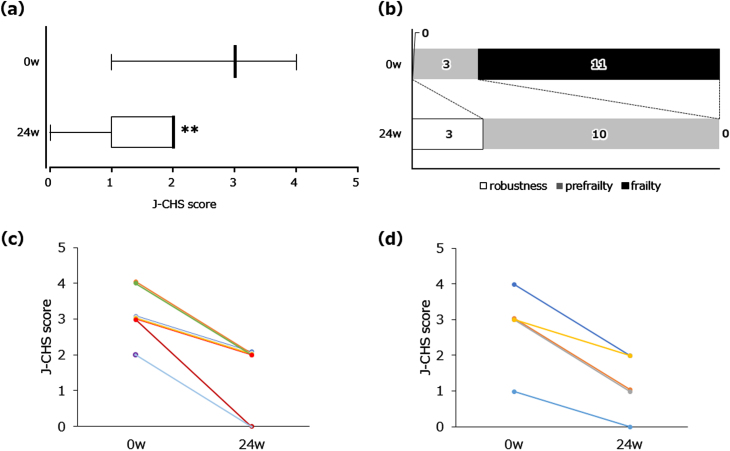
Changes in J-CHS score after NYT treatment. Frailty: 3 or more are satisfied; prefrailty: 1 or 2 criteria are met; robustness: none of the criteria are met. Box plot (a) showing median, quartiles, and bars indicating the maximum and minimum values; *n* = 14 (0 week (w)), *n* = 13 (24 w); ^**^p < 0.01 versus 0 w (Wilcoxon signed-rank test). The transition of frailty states (b). White, gray, and black squares indicate robustness, prefrailty, and frailty, respectively. Line graphs showing all individual data in MCI (c) and mild AD (d), separately. *n* = 9 (MCI), *n* = 5 (mild AD).

As shown in Fig. 2a, a significant decrease in the J-CHS score was observed (baseline: 3.0 [3.0-3.0]; 24 weeks: 2.0 [1.0-2.0], *p* < 0.01) after NYT treatment for 24 weeks. As mentioned above, 11 patients with frailty and 3 with prefrailty were enrolled. By week 24, frailty was no longer observed as all patients were restored to prefrailty condition or robustness (Fig. 2b). Figure 2c and 2d depict the changes in individual J-CHS scores of patients with MCI and mild AD, respectively. J-CHS scores were reduced in all patients without diagnostic distinction as to MCI or mild AD status. Among the five physical components (unintentional weight loss, low grip strength, self-reported exhaustion, slow walking speed, and low physical activity) of the J-CHS criteria, self-reported exhaustion was especially ameliorated by NYT (data not shown). The VAS score for fatigue decreased significantly at week 16 (Table 2). No significant changes were observed in body weight, lean body mass, fat mass, grip strength, walking speed, or CONUT score. However, walking speed at week 24 showed a tendency to increase from baseline (0.821±0.305) to 24 weeks (0.885±0.301) (*p* < 0.1). The NPI subcategory scores for ‘depression’ significantly decreased at weeks 8 and 16. The minimum and maximum values at weeks 0, 4, 8, 16, and 24 were 0.0–3.0, 0.0–4.0, 0.0–2.0, 0.0–2.0, and 0.0–4.0, respectively (data not shown). NPI subcategory scores for ‘apathy’ showed no significant change (Table 2). As shown in Table 3, cognitive function assessed by the global CDR, CDR-SB, and MoCA-J total scores showed no significant decrease and remained at the baseline level throughout the 24-week study period.

**Table 2:** Changes in physical/mental frailty assessments

	0w	4w	8w	16w	24w
Body weight, kg	47.5±9.5	47.8±9.8	47.4±9.3	47.7±9.7	49.0±9.3
Lean body mass, kg	31.9±5.8	31.9±6.0	31.6±5.6	32.0±6.2	32.8±6.9
Fat mass, kg	13.6±5.0	13.9±5.0	13.8±5.1	13.6±4.9	15.0±4.6
Grip strength, kg	20.8±6.7	–	–	–	22.6±7.4
Walking speed, m/sec	0.821±0.305	–	–	–	0.885±0.301^†^
Fatigue VAS, mm	40.5 (30.0–51.5)	38.5 (25.3–49.3)	22.0 (18.5–41.3)^†^	25.0 (20.3–29.5)*	33.0 (19.0–48.0)
CONUT score	0.5 (0.0-1.0)	1.0 (0.0–1.8)	1.0 (0.3–2.0)	1.0 (0.0–2.0)	1.0 (0.0–2.0)
Serum albumin, g/dL	4.1±0.3	4.1±0.3	4.1±0.2	4.0±0.2^†^	4.1±0.3
Total lymphocyte count, /μL	1848.1±752.2	1703.9±685.8	1611.8±636.9	1631.0±729.5	1518.8±512.2
Total cholesterol level, mg/dL	206.7±35.8	204.1±29.6	200.1±31.6	206.5±33.8	202.3±28.9
Depression (NPI subcategory score)	1.0 (0.0-1.0)	0.0 (0.0–0.8)	0.0 (0.0–0.8)*	0.0 (0.0-0.0)*	0.0 (0.0-0.0)
Apathy (NPI subcategory score)	0.0 (0.0–1.8)	0.0 (0.0–1.8)	0.0 (0.0–0.8)	0.5 (0.0–2.0)	0.0 (0.0–2.0)

The data are presented as mean±SD or median with quartiles in parentheses. ^*^p < 0.05, ^†^*p* < 0.1 versus 0 w (Wilcoxon signed-rank test or paired *t*-test). *n* = 14 (0, 4, 8 and 16 weeks (w)), *n* = 13 (24 w). CONUT, controlling nutritional status; NPI, Neuropsychiatric Inventory.

**Table 3:** Changes in cognitive function

	0w	24w
CDR
Global CDR	0.5 (0.5-0.5)	0.5 (0.5–1.0)
Sum of Boxes	3.5 (2.6–4.0)	3.0 (2.0–5.0)
Memory	0.8 (0.5–1.0)	1.0 (0.5–1.0)
Orientation	0.5 (0.5–0.9)	0.5 (0.5-1.0)
Judgement and Problem Solving	0.5 (0.5–1.0)	0.5 (0.5–1.0)
Community Affairs	0.5 (0.5-0.5)	0.5 (0.5–1.0)
Home and Hobbies	0.5 (0.1–0.5)	0.5 (0.5–1.0)
Personal Care	0.0 (0.0-1.0)	0.0 (0.0-0.0)
MoCA-J total score	18.0 (14.3–20.8)	18.0 (17.0–19.0)
Executive and visuospatial	3.5 (2.3–4.8)	3.0 (3.0-4.0)
Naming	3.0 (1.3–3.0)	2.0 (1.0–3.0)^†^
Attention	5.0 (4.0–5.8)	4.0 (3.0–5.0)
Language	1.0 (1.0–1.8)	1.0 (1.0-1.0)
Abstraction	1.0 (1.0-2.0)	1.0 (1.0-2.0)
Delayed recall	0.0 (0.0-0.0)	0.0 (0.0-1.0)
Orientation	5.0 (4.3–5.8)	5.0 (4.0–6.0)

Data are presented as medians, with quartiles in parentheses. ^†^*p* < 0.1 versus 0 w (Wilcoxon signed-rank test). *n* = 14 (0 week (w)), *n* = 13 (24 w). CDR, Clinical Dementia Rating; MoCA, Montreal Cognitive Assessment.

### Adverse events

A serious adverse event—a fracture from falling—was noted in one patient (hospitalization; continuation). Non-serious adverse events were noted in five patients: pyrexia (continuation), Meniere’s disease (continuation), subgaleal hematoma, left forearm epidermis detachment, left thigh contusion and edema (continuation), and two patients had only edema (continuation in both cases). A causal relationship with NYT was excluded for all adverse events, except for one patient with edema.

## DISCUSSION

In the pathophysiology of frailty [7], the initial lifelong accumulation of molecular and cellular damage caused by multiple mechanisms regulated by a complex maintenance and repair network under the influence of genetic, environmental, and epigenetic mechanisms results in a gradual decline in physiological reserve following stress in multiple organs, such as the brain, skeletal muscle, immune system (inflammatory cytokines), and endocrine system. This reduced physiological reserve involves low physical activity (fatigue and low muscle strength) and malnutrition (anorexia and weight loss) that may be caused by mitochondrial dysfunction [31], oxidative stress [32], and negative energy homeostasis with aging, and finally declines into frailty, which may be associated with MCI [14] or dementia [15]. Buchman et al., using a continuous composite measure of frailty, documented its progressive rate of change in older persons [33] and found that accumulation of common brain pathologies, including AD pathology and loss of neurons in the substantia nigra relating to the reward system, contributed to progression of age-related physical frailty [34]. As such, in frail patients including those with anorexia with brain pathologies, self-improvement without therapeutic intervention would not be expected.

The primary outcome score for ‘anorexia’ significantly improved by week 4 and continued throughout the observation period of NYT treatment in prefrail or frail MCI or mild AD patients. To the best of our knowledge, this is the first study to explore the effects of NYT on anorexia in frail older patients with MCI or mild dementia.

Neuropeptide Y (NPY) and ghrelin are the most potent central and peripheral inducers of appetite, respectively. NYT directly targets both ghrelin-responsive and unresponsive NPY neurons in the hypothalamic arcuate nucleus, a key hypothalamic nucleus in appetite regulation, and preserves food intake and body weight in cisplatin-treated anorectic mice [35]. Using an *in vitro* assay system to observe the activities of several G-protein-coupled receptors, Miyano et al. reported that NYT and its constituent, Citrus Unshiu Peel, could increase food intake via activation of orexin 1 receptor-expressing neurons in the hypothalamus [36]. Moreover, NYT promotes feeding and reduces activity to conserve energy under conditions of negative energy homeostasis, such as aging and frailty. These effects are partly mediated by signaling through the NPY system [37].

Another study on the anti-anorexic effect of NYT showed activation of dopamine D2 receptors [38]. The inhibitory effects of NYT ingredients on dopamine metabolism-related enzymes, such as monoamine oxidase B, catechol-O-methyl transferase, and dopamine transporters were also determined in their study. Miyazaki et al. demonstrated that NYT increased dopamine content in a cell culture model [39]. The mesolimbic dopaminergic pathway is known to play an important role in feeding and the motivation/reward system [40]. Electrophysiological examination showed that NYT might enhance stimulus-dependent responsiveness to medium-sized spiny neurons in the core and shell regions of the nucleus accumbens [41].

After a 24-week observation period, the severity of frailty evaluated using the J-CHS criteria, one of the secondary outcome measures, was ameliorated in NYT-treated patients. Regarding the frailty-related indices, the average fatigue score by the fatigue VAS showed a transient and significant decrease at week 16; the average walking speed tended to increase at week 24; the average body weight, fat mass, and grip strength increased slightly at week 24, but not significantly. The average lean body mass did not change during the 24-week observation period.

Fatigue is associated with mitochondrial dysfunction [42], and NYT has been reported to increase mitochondrial content and function in human umbilical vein endothelial cells (HUVECs) [43]. Ginseng, a component of NYT, is widely used for treating fatigue because it has been reported to improve energy, physical and emotional health, and well-being [44]. Ginsenoside Rg1, the main active ingredient in Ginseng, has been reported to have anti-fatigue effects by regulating the number of T cell subsets and enhancing immunity in rats with chronic fatigue syndrome [45]. Furthermore, one of the constituents of NYT, Schisandra Fruit, has been reported to improve endurance and energy metabolism in exercised rats and has the potential to upregulate peroxisome proliferator-activated receptor-gamma coactivator-1 α in skeletal muscle, which is a key regulator of energy metabolism [46]. The anti-fatigue effect of NYT found in the present study may be correlated with improvement in mitochondrial function, the immune system, and energy metabolism, which are associated with the pathophysiology of frailty.

The mechanism underlying the tendency to increased walking speed was unclear; however, it might be induced by an alteration in muscle quality. Matsubara et al. showed that NYT improved forelimb grip strength in old mice compared to that in the control group, without affecting skeletal muscle loss. These results suggest that NYT may alter muscle quality rather than increase muscle mass. In addition, a cell-free assay revealed that the antioxidant effects of NYT might be associated with the improvement of frailty-like symptoms in their study [47]. We also suggest that the slight increases in body weight and fat mass at week 24 might be caused by an early improvement in anorexia beginning at week 4. These changes would benefit patients by counteracting aging through circulating extracellular nicotinamide phosphoribosyltransferase (eNAMPT), which promotes systemic NAD+ biosynthesis, and is produced by adipose tissue [48].

This study showed that NYT statistically improved ‘depression’ transiently at weeks 8 and 16 in spite of low basal scores, which were assessed and evaluated by the NPI depression score. Similarly, NYT has been reported to improve cognitive function and reduce depression in patients with AD when added to donepezil treatment [18]. In the frail brain, neurons with high metabolic demands, such as hippocampal pyramidal neurons and microglial cells, may be disproportionally affected by aging, in terms of altered synaptic function, protein transport, and mitochondrial activity (energy failure) [7]. Cognitive decline [49] and depression [50] are linked to changes in cellular structure and function. NYT has been reported to inhibit microglial activation in the hippocampus [51], which may partially account for the antidepressant effects of NYT in the present study.

Although there have been reports in the past that NYT was effective for apathy in frail AD patients [21], the NPI ‘apathy’ score showed no significant change after treatment with NYT for 24 weeks in the present study. The scoring of apathy was not included in the inclusion criteria, which may have led to the result that the basal apathy score at week 0 was quite low in the present study. Differences in the case registration methods may explain the contradictory results between the present and previous studies. No significant changes were observed in CONUT scores at week 24 in the present study, which can be used to assess a patient’s nutritional status. There is a possibility that improvements in anorexia and frailty may not necessarily be reflected in blood nutrition indices because of the age-related reduction in absorption from epithelial cells in the small intestinal villi [52].

Frailty is associated with cognitive decline [13] and AD [53]. Wallace et al. demonstrated that frailty influenced the odds of cognitive impairment and dementia, suggesting that interventions for frailty may be useful for the prevention of disease at any stage of neuropathological accumulation [54]. In the present study, the cognitive function and dementia staging, evaluated by MoCA-J and CDR, were maintained at baseline levels for the 24 weeks of the NYT treatment period (only one patient with mild AD had donepezil in this study, while the other patients received no therapeutic drugs for dementia). Since MCI shows a more rapid rate of cognitive decline [1], these results suggest that NYT might have a disease progression inhibitory or preventive effect on conversion to dementia through beneficial effects on frailty-related factors, especially anorexia and fatigue. As described in the Introduction, NYT reversed age-induced demyelination, leading to an acceleration of axonal remyelination in the aged mouse brain [17]. Furthermore, it was reported that NYT activated choline acetyltransferase and induced nerve growth factor secretion from cultured rat astrocytes [55], suggesting that NYT may have potential therapeutic efficacy in the treatment of AD patients. In a recent report, the combination of a decline in both gait speed and memory had the strongest association with dementia risk [56]. We hypothesized that maintaining cognitive function and a tendency to increased walking speed by NYT treatment would be beneficial for the prognosis of dementia. However, further long-term interventional studies of at least one year may be required to reliably demonstrate such preventive effects on other frailty-related factors, including walking speed, body weight, body composition, and grip strength.

In conclusion, the results suggest that NYT may be safe and effective in the treatment of frailty, especially for anorexia and fatigue, in both MCI and mild AD patients, which would be beneficial for the prognosis of dementia. However, the present study has several limitations. First, it was an open-label, non-controlled study; therefore, the reliability of our results might not be high. Additionally, the sample size was small. Thus, in the future it will be necessary to conduct a randomized controlled trial or double-blind placebo-controlled study on a larger number of patients to confirm these results.
